# A Novel Method to Determine the Thermal Conductivity of Interfacial Layers Surrounding the Nanoparticles of a Nanofluid

**DOI:** 10.3390/nano4040844

**Published:** 2014-10-13

**Authors:** Rajinder Pal

**Affiliations:** Department of Chemical Engineering, University of Waterloo, Waterloo, ON N2L 3G1, Canada; E-Mail: rpal@uwaterloo.ca; Tel.: +1-519-888-4567 (ext. 32985)

**Keywords:** nanoparticle, nanofluid, thermal conductivity, interfacial layer, nanolayer

## Abstract

Nanofluids are becoming increasingly popular as heat transfer fluids in a variety of industrial applications, due to their enhanced heat transfer characteristics. The thermal conductivity of nanofluids is usually found to be much larger than that predicted from the classical models, such as the Maxwell model. The key mechanism of enhancement of thermal conductivity of dilute nanofluids is the solvation of nanoparticles with a layer of matrix liquid. As of now, little is known quantitatively about the thermal conductivity of the interfacial layers surrounding the nanoparticles. In this article, a novel method is presented to determine the thermal conductivity of the interfacial layers of the nanoparticles. The proposed method allows the estimation of the thermal conductivity of interfacial layers based on the combined measurements of the intrinsic viscosity and intrinsic thermal conductivity of a bulk nanofluid. From the measured intrinsic viscosity of the nanofluid, the thickness of the interfacial layer is estimated. Using the known interfacial layer thickness along with the measured intrinsic thermal conductivity of the nanofluid, the thermal conductivity of the interfacial layer is estimated. The proposed method is validated by simulation and experimental results.

## 1. Introduction

Nanofluids are engineered suspensions of fine nanometer-sized particles in a base fluid [1]. They are known to exhibit enhanced thermophysical properties, such as thermal conductivity and viscosity, even at a very low concentration of nanoparticles. The nanoparticles of nanofluids are often stabilized with the help of additives, such as a surfactant or polymer. The surfactant or polymer molecules adsorb at the surface of the nanoparticles and, hence, provide steric-stabilization to them. Nanofluids play an important role in a variety of applications, such as paints, coatings and pharmaceutical formulations [2]. Due to their enhanced thermal conductivity and heat transfer characteristics, nanofluids are gaining popularity as heat transfer fluids in a variety of industrial applications [3,4,5,6,7,8,9,10,11,12]. Oil, water and ethylene glycol are often used as the base fluids in the manufacturing of heat transfer nanofluids. The nanoparticles are usually made of metals, oxides or carbides.

In dilute nanofluids, the main mechanism of enhancement of the thermal and physical properties of nanofluids is the solvation of nanoparticles with a layer of matrix fluid [13,14]. The physical properties (such as thermal conductivity) of the fluid layer immobilized at the surface of the particles are significantly different from that of the bulk matrix fluid. It is of practical and theoretical interest to estimate the thermal conductivity of the interfacial or solvation layers surrounding the nanoparticles of a nanofluid. As of now, little is known quantitatively about the thermal conductivity of the interfacial layers. In this article, we present a novel approach to estimate the thermal conductivity of interfacial layers using experimental measurements of the viscosity and thermal conductivity of dilute nanofluids. To our knowledge, this is the first study to present a methodology to estimate the thermal conductivity of the interfacial layers surrounding the nanoparticles of a nanofluid.

## 2. Theoretical Background

### 2.1. Viscosity of Nanofluids

The viscosity of dilute Newtonian suspensions of solid spherical particles is given by the following Einstein viscosity equation [15,16]:

(1) $${\text{η}}_{\text{r}}=1+2.5\text{φ}$$

where η_r_ is the relative viscosity of a suspension defined as the ratio of suspension viscosity to the base fluid viscosity and φ is the volume fraction of particles. The Einstein equation is accurate only for small values of φ, on the order of 2% or so [17]. However, even very dilute nanofluids with φ ≤ 0.02 do not obey the Einstein equation. The Einstein equation severely underpredicts the nanofluid viscosity. It has been shown by Pal [14] that the viscosity of nanofluids, in general, is strongly influenced by the “solvation” and “clustering” of nanoparticles. The thermal conductivity of nanofluids is also significantly affected by the “solvation” and “clustering” of nanoparticles [18]. In very dilute nanofluids, however, only the “solvation effect” is present with negligible or no clustering of nanoparticles. Due to solvation of particles, the effective volume fraction of the dispersed-phase (φ_eff_) is significantly larger than the actual volume fraction (φ) of nanoparticles (un-solvated). The relationship between φ_eff_ and φ is given as:

(2) $${\text{φ}}_{\text{eff}}={\left(\frac{R}{R_{\text{o}}}\right)}^{3}\text{φ}={\left(1+\frac{\text{δ}}{R_{\text{o}}}\right)}^{3}\text{φ}=k_{\text{s}}\text{φ}$$

where *R* is the radius of the composite (solid core plus solvation layer) nanoparticle; *R*_o_ is the radius of the dry (un-solvated) nanoparticle; δ is the thickness of the interfacial solvation layer; and *k*_s_ is the solvation coefficient. Note that *k*_s_ = (1 + δ/*R*_o_)^3^.

The intrinsic viscosity [η] of a nanofluid is defined as:

(3) $${[\text{η}]\;=Lim}_{\text{φ}\to 0}\left(\frac{{\text{η}}_{\text{r}}-1}{\text{φ}}\right)$$

Thus, for infinitely dilute nanofluids, one can write:

(4) $${\text{η}}_{\text{r}}=1+\left[\text{η}\right]\text{φ}$$

For suspensions of solvated nanoparticles, the Einstein equation gives:

(5) $${\text{η}}_{\text{r}}=1+2.5{\text{φ}}_{\text{eff}}=1+2.5(k_{\text{s}}\text{φ})$$

Upon comparison of Equations (4) and (5), it follows that:

(6) $$[\text{η}]=2.5k_{\text{s}}$$

The intrinsic viscosity of a nanofluid is accessible through experimental viscosity data on dilute nanofluids. It can be obtained from the slope of η_r_ *versus* φ plot at φ → 0 (φ ≤ 0.02). Alternatively, [η] can be determined from the slope of 1/ η_r_ *versus* φ plot at φ → 0. Note that the Einstein equation could be re-written as:

(7) $$\frac{1}{{\text{η}}_{\text{r}}}=\frac{1}{1+[\text{η}]\text{φ}}={\left(1+[\text{η}]\text{φ}\right)}^{-1}=1-[\text{η}]\text{φ}+........$$

The slope of 1/ η_r_ *versus* φ data in the limit of φ → 0 is −[η]. Once the intrinsic viscosity is known, the solvation coefficient *k*_s_ of a nanofluid can be determined from Equation (6).

### 2.2. Thermal Conductivity of Nanofluids

For an infinitely dilute suspension of spherical particles, the exact expression for the thermal conductivity is given as [19]:

(8) $$\frac{K}{K_{\text{m}}}=1+3\left(\frac{K_{\text{d}}-K_{\text{m}}}{K_{\text{d}}+2K_{\text{m}}}\right)\text{ φ}$$

where *K* is the thermal conductivity of the suspension; *K*_m_ is the thermal conductivity of the matrix; *K*_d_ is the thermal conductivity of particles and φ is the volume fraction of particles. This equation could be re-written as:

(9) $$K_{\text{r}}=1+3\left(\frac{\text{λ}-1}{\text{λ}+2}\right)\text{ φ}$$

where *K*_r_ is the relative thermal conductivity defined as *K* / *K*_m_; and λ is the thermal conductivity ratio defined as *K*_d_ / *K*_m_. Equation (9) follows from the following Maxwell–Eucken equation for the thermal conductivity of suspensions [20]:

(10) $$K_{\text{r}}=\frac{K}{K_{\text{m}}}=\left[\frac{1+2\text{φ }\left(\frac{\text{λ}-1}{\text{λ}+2}\right)}{1-\text{φ}\left(\frac{\text{λ}-1}{\text{λ}+2}\right)}\right]$$

In the limit φ → 0, Equation (10) reduces to Equation (9). Equations (8)–(10) can be applied to nanofluids only under the condition that the nanoparticles are un-solvated. As nanoparticles of nanofluids are known to undergo significant solvation, the thermal conductivities predicted by Equation (8) or (9) are usually much lower than the experimental values. The enhancement of thermal conductivity of nanofluids is due to the formation of structured nanolayers of matrix liquid molecules on the surface of the solid particles [13,21,22,23]. The molecules of the matrix liquid in the nanolayers surrounding the particles are in a physical state intermediate between a bulk matrix liquid and a solid, and therefore, the solvation nanolayers possess a thermal conductivity significantly higher than that of the bulk matrix liquid [23]. The presence of surfactant (stabilizer) molecules at the interface can also affect the properties of the interfacial layer. The temperature is also known to influence the surface and interfacial layer properties of the nanoparticles [24].

The solvated nanoparticles of nanofluids are essentially core-shell-type nanoparticles consisting of a solid core of thermal conductivity *K*_3_, a shell (solvated layer) of thermal conductivity *K*_2_ and a matrix fluid of thermal conductivit *K*_1_. The exact expression for the relative thermal conductivity of an infinitely dilute suspension of core-shell particles is given as [25]:

(11) $$K_{\text{r}}=\frac{K}{K_{1}}=1+3\left[\frac{\left(K_{3}-K_{2}\right)\left(2K_{2}+K_{1}\right)+k_{\text{s}}\left(K_{3}+2K_{2}\right)\left(K_{2}-K_{1}\right)}{\left(K_{3}+2K_{2}\right)\left(K_{2}+2K_{1}\right)+\left(2/k_{\text{s}}\right)\left(K_{3}-K_{2}\right)\left(K_{2}-K_{1}\right)}\right]\text{ φ}$$

where φ is the volume fraction of un-solvated particles and *k*_s_ is the solvation coefficient, defined earlier as *k*_s_ = (1 + δ/*R*_o_)^3^, where *R*_o_ is the un-solvated particle radius and δ is the interfacial nanolayer thickness. Equation (11) is accurate for small values of φ, on the order of 5% or so [25], and it could be expressed in terms of φ_eff_ (= *k*_s_φ) as:

(12) $$K_{\text{r}}=\frac{K}{K_{1}}=1+3\left[\frac{\left(K_{3}-K_{2}\right)\left(2K_{2}+K_{1}\right)+k_{\text{s}}\left(K_{3}+2K_{2}\right)\left(K_{2}-K_{1}\right)}{2\left(K_{3}-K_{2}\right)\left(K_{2}-K_{1}\right)+k_{\text{s}}\left(K_{3}+2K_{2}\right)\left(K_{2}+2K_{1}\right)}\right]\;{\text{φ}}_{\text{eff}}$$

Another useful form of Equation (12) is as follows:

(13) $$K_{\text{r}}=\frac{K}{K_{1}}=1+3\left[\frac{\left(2+k_{\text{s}}\right){\text{λ}}_{31}-2\left(1-k_{\text{s}}\right){\text{λ}}_{21}-\left\{1+2k_{\text{s}}-\left(1-k_{\text{s}}\right){\text{λ}}_{32}\right\}}{\left(2+k_{\text{s}}\right){\text{λ}}_{31}-2\left(1-k_{\text{s}}\right){\text{λ}}_{21}+2\left\{1+2k_{\text{s}}-\left(1-k_{\text{s}}\right){\text{λ}}_{32}\right\}}\right]\;{\text{φ}}_{\text{eff}}$$

where λ_31_ = *K*_3_ / *K*_1_, λ_21_ = *K*_2_ / *K*_1_ and λ_32_ = *K*_3_ / *K*_2_. Upon further rearrangement, Equation (13) could be expressed as:

(14) $$K_{\text{r}}=\frac{K}{K_{1}}=1+3\left(\frac{\left(B/A\right)-1}{\left(B/A\right)+2}\right)\;{\text{φ}}_{\text{eff}}$$

where *B* and *A* are given as:

(15) $$B=(2+k_{\text{s}})\frac{K_{3}}{K_{1}}-2(1-k_{\text{s}})\frac{K_{2}}{K_{1}}$$

(16) $$A=1+2k_{\text{s}}-(1-k_{\text{s}})\frac{K_{3}}{K_{2}}$$

In the absence of any solvation, *k*_s_ = 1, *B* = 3(*K*_3_ / *K*_1_), *A* = 3, φ_eff_ = φ, and consequently, Equation (14) reduces to Equation (8). Note that *K*_d_ = *K*_3_ and *K*_m_ = *K*_1_.

Upon comparison of Equations (14) with (9), the ratio *B* / *A* could be interpreted as the ratio of effective thermal conductivity of the core-shell particle (*K*_d,core-shell_) to the matrix thermal conductivity, that is:

(17) $$\frac{K_{\text{d,core-shell}}}{K_{1}}=\frac{B}{A}=\frac{(2+k_{\text{s}})\frac{K_{3}}{K_{1}}-2(1-k_{\text{s}})\frac{K_{2}}{K_{1}}}{1+2k_{\text{s}}-(1-k_{\text{s}})\frac{K_{3}}{K_{2}}}$$

The intrinsic thermal conductivity [*K*] of a nanofluid is defined as:

(18) $${[K]\;=Lim}_{\text{φ}\to 0}\left(\frac{K_{\text{r}}-1}{\text{φ}}\right)$$

where φ s the volume fraction of un-solvated (that is, core) particles. For infinitely dilute nanofluids, one can write:

(19) $$K_{\text{r}}=1+\left[K\right]\text{φ}$$

The intrinsic thermal conductivity of a nanofluid is accessible through experimental thermal conductivity data on dilute nanofluids. It can be obtained from the slope of *K*_r_ *versus* φ plot at φ → 0 (φ < 0.05). Alternatively, [*K*] can be determined from the slope of 1/ *K*_r_ *versus* φ plot at φ → 0. Note that Equation (19) could be re-written as:

(20) $$\frac{1}{K_{\text{r}}}=\frac{1}{1+[K]\text{φ}}={\left(1+[K]\text{φ}\right)}^{-1}=1-[K]\text{φ}+........$$

The slope of 1/ *K*_r_ *versus* φ data in the limit of φ → 0 is −[*K*].

## 3. Estimation of Interfacial Layer Thermal Conductivity

The thermal conductivity of interfacial layer surrounding the nanoparticles of a nanofluid can be determined using the combined measurements of the viscosity and thermal conductivity of very dilute nanofluids.

Upon comparing Equations (14) and (19), the intrinsic thermal conductivity can be expressed as:

(21) $$[K]=3\left(\frac{\left(B/A\right)-1}{\left(B/A\right)+2}\right)\left(\frac{{\text{φ}}_{\text{eff}}}{\text{φ}}\right)$$

As the ratio φ_eff_ / φ is the solvation coefficient *k*_s_, Equation (21) becomes:

(22) $$[K]=3\left(\frac{\left(B/A\right)-1}{\left(B/A\right)+2}\right)k_{\text{s}}$$

where *B* and *A* are defined in Equations (15) and (16).

From the experimentally determined values of intrinsic viscosity [η] and intrinsic thermal conductivity [*K*], one can estimate the thermal conductivity of the interfacial layer (that is, *K*_2_) from Equation (22). Equation (22) could be re-cast as follows:

(23) $$[K]=3\left(\frac{\frac{(2+k_{\text{s}})\frac{K_{3}}{K_{1}}-2(1-k_{\text{s}})\frac{K_{2}}{K_{1}}}{1+2k_{\text{s}}-(1-k_{\text{s}})\frac{K_{3}}{K_{2}}}-1}{\frac{(2+k_{\text{s}}\text{)}\frac{K_{3}}{K_{1}}-2(1-k_{\text{s}})\frac{K_{2}}{K_{1}}}{1+2k_{\text{s}}-(1-k_{\text{s}})\frac{K_{3}}{K_{2}}}+2}\right)k_{\text{s}}$$

The only unknown in Equation (23) is the interfacial-layer thermal conductivity *K*_2_, as all other quantities ([*K*], *k*_s_, *K*_1_ and *K*_3_) are known. Note that the solvation coefficient *k*_s_ is known from the intrinsic viscosity through Equation (6). In the absence of any solvation of particles, *k*_s_ = 1, and the intrinsic thermal conductivity [*K*] reduces to:

(24) $$[K]=3\left[\frac{\frac{K_{3}}{K_{1}}-1}{\frac{K_{3}}{K_{1}}+2}\right]$$

Equation (23) is a quadratic equation that can be expressed as:

(25) $$aK_{2}^{2}+bK_{2}+c=0$$

where:

(26) $$a=\frac{2\left(1-k_{\text{s}}\right)}{K_{1}}\left(3k_{\text{s}}-[K]\right)$$

(27) $$b=\left[\left(2+k_{\text{s}}\right)\;\frac{K_{3}}{K_{1}}+2\left(1+2k_{\text{s}}\right)\right]\;[K]-\left[3\left(2+k_{\text{s}}\right)\frac{K_{3}}{K_{1}}-3\left(1+2k_{\text{s}}\right)\right]k_{\text{s}}$$

(28) $$c=-\left(1-k_{\text{s}}\right)K_{3}\left[3k_{\text{s}}+2[K]\right]$$

Thus *K*_2_ can be calculated from the following expression:

(29) $$K_{2}=\frac{-b\pm \sqrt{b^{2}-4ac}}{2a}$$

## 4. Simulation Results

Consider the case where *K*_1_ = 0.6 *W* / *m* ⋅*K*, *K*_3_ = 12 *W* / *m* ⋅*K*, [η] = 5 and [*K*] = 4. In this case, the enhancement of the thermal conductivity of the nanofluid is more than that predicted from the Maxwell equation (Equation (24)), which gives [*K*] of 2.59. With the given values of *K*_1_, *K*_3_, [η], and [*K*], we obtain the following results: *k*_s_ = 2, *a* = −6.66667, *b* = −90, *c* = 168, *K*_2_ = 1.662 *W* / *m* ⋅*K* and *K*_2_ / *K*_1_ = 2.77. Thus, the thermal conductivity of the interfacial layer of nanoparticles is 2.77-times the thermal conductivity of the base (matrix) fluid.

Consider another example, where *K*_1_ = 0.258 *W* / *m* ⋅*K*, *K*_3_ = 400 *W* / *m* ⋅*K*, [η] = 10 and [*K*] = 8. In this case, the enhancement of thermal conductivity of nanofluid is much more than that predicted from the Maxwell equation (Equation (24)), which gives a [*K*] of 2.994. With the given values of *K*_1_, *K*_3_, [η], and [*K*], we obtain the following results: *k*_s_ = 4, *a* = −93.023, *b* = −36,957.3, *c* = 33,600, *K*_2_ = 0.907254 *W* / *m* ⋅*K* and *K*_2_ / *K*_1_ = 3.52. Thus, the thermal conductivity of the interfacial layer of nanoparticles is 3.52-times the thermal conductivity of the base (matrix) fluid.

## 5. Experimental Validation

As already noted, knowledge of the following quantities is required in order to evaluate the thermal conductivity *K*_2_ of the interfacial layer of nanoparticles: *K*_1_, *K*_3_, [η], and [*K*]. For a given nanofluid, *K*_1_ (base fluid) and *K*_3_ (unsolvated nanoparticles) are known. The determination of intrinsic viscosity [η] requires experimental viscosity data on a dilute nanofluid at low values of nanoparticle concentration. Likewise the determination of intrinsic thermal conductivity [*K*] requires experimental thermal conductivity data on a dilute nanofluid at low values of nanoparticle concentration.

**Figure 1:**
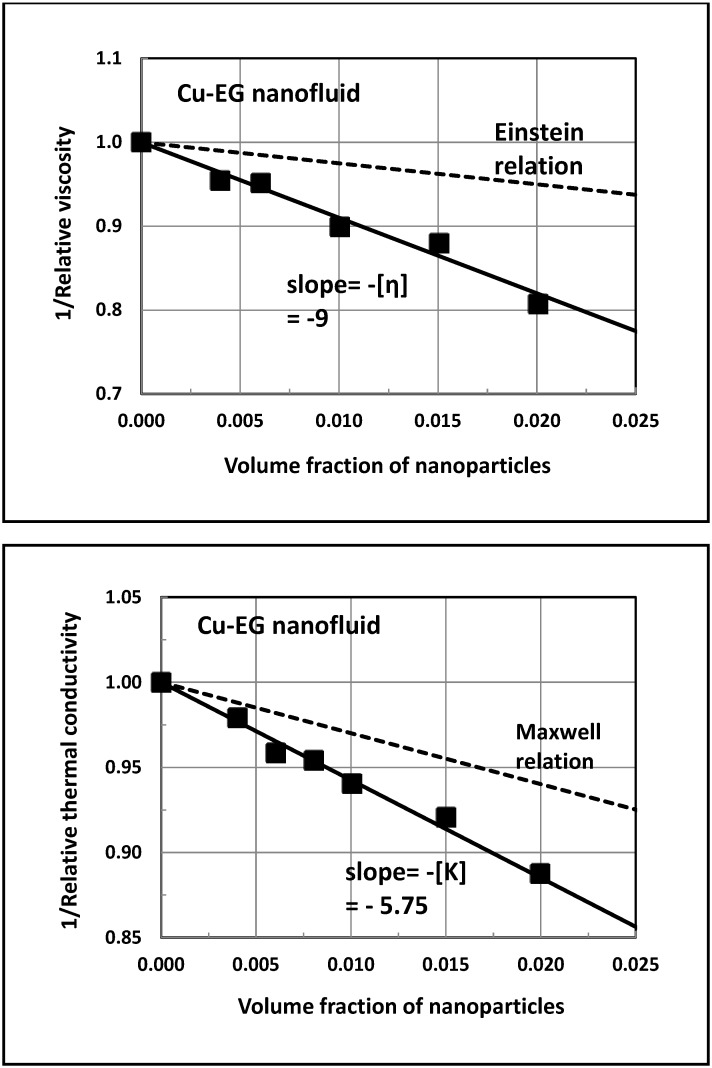
Estimation of [η] and [*K*] for the copper—ethylene glycol nanofluid.

Figure 1 shows the plots of the relative viscosity and relative thermal conductivity for dilute copper—ethylene glycol nanofluid (referred to as Cu-EG nanofluid), based on the experimental data of Garg *et al.* [26]. The properties are measured at 25 °C. The un-solvated copper nanoparticles are 200 nm in diameter, and the values of *K*_1_ and *K*_3_ are 0.258 and 400 *W* / *m* ⋅*K*, respectively. The data are plotted as 1/ η_r_ *versus* φ and as 1/ *K*_r_ *versus* φ. The plots are linear over the φ range of 0 to 0.02. The slope of 1/ η_r_ *versus* φ plot is −9 and the slope of 1/ *K*_r_ *versus* φ plot is −5.75. It should be noted that these nanofluids are Newtonian in nature with the negligible dependence of viscosity on the shear rate. The shear rate range covered in the experiments is 3–3,000 s^−1^. The linear dependence of η_r_ on φ and the lack of shear dependence of viscosity (Newtonian behavior) suggest that clustering of nanoparticles is negligible in these nanofluids over the φ range of 0 to 2%. The clustering of nanoparticles is expected to impart non-Newtonian shear-thinning behavior to nanofluids [14]. The dependence of η_r_ on φ is also expected to be non-linear [14].

According to Figure 1, the values of [η] and [*K*] for the Cu-EG nanofluid are 9 and 5.75, respectively. Equation (24) gives a [*K*] of 2.994. Obviously, the actual enhancement of thermal conductivity of nanofluid ([*K*] = 5.75) is substantially larger than that predicted from the Maxwell equation (Equation (24)). For the given values of *K*_1_, *K*_3_, [*η*], and [*K*], we obtain the following results: *k*_s_ = 3.6, *a* = −101.783, *b* = −43,662.1, *c* = 23,192, *K*_2_ = 0.5305 *W* / *m* ⋅*K* and *K*_2_ / *K*_1_ = 2.056. Thus, the thermal conductivity of the interfacial layer of copper nanoparticles in Cu-EG nanofluid is 2.056-times that of the thermal conductivity of the base (matrix) fluid.

**Figure 2:**
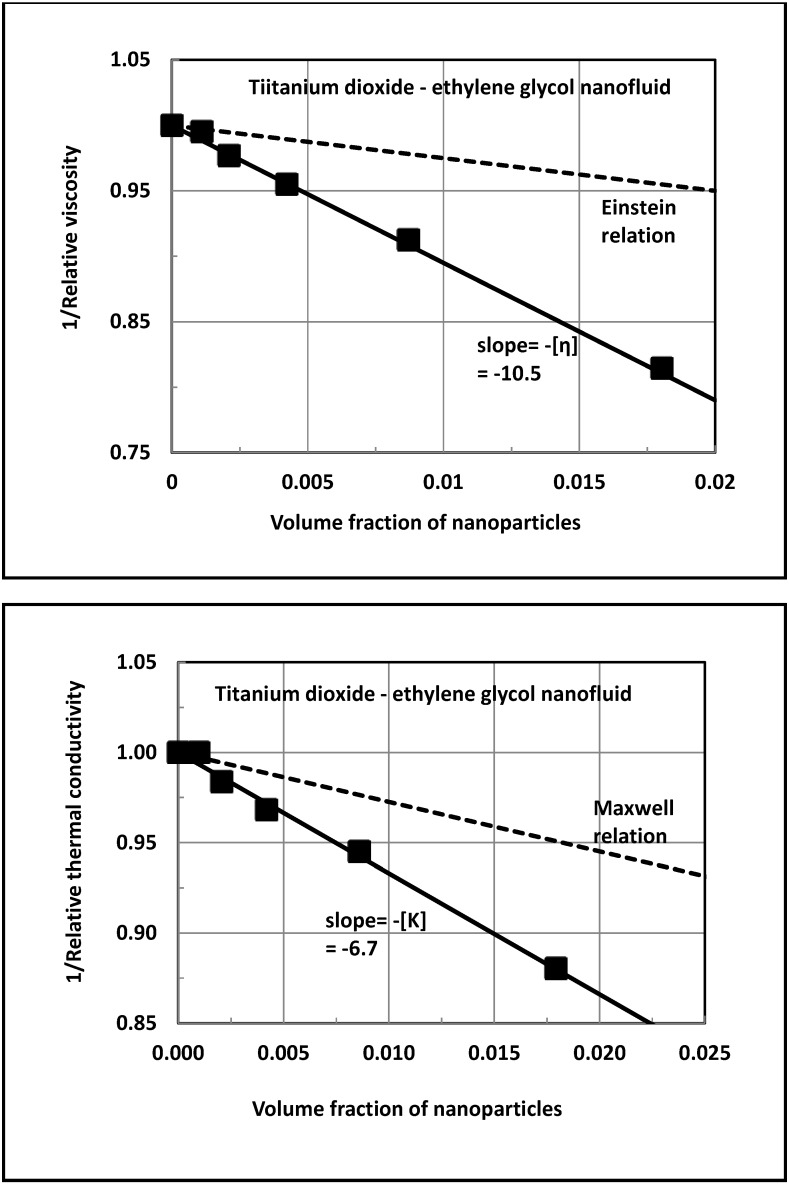
Estimation of [η] and [*K*] for titanium dioxide—ethylene glycol nanofluid.

Figure 2 shows the plots of the relative viscosity and relative thermal conductivity for dilute titanium dioxide—ethylene glycol nanofluid (referred to as TiO_2_-EG nanofluid), based on the experimental data of Chen *et al.* [11]. The thermal conductivity is measured at 20 °C, and the viscosity is measured over a temperature range of 20–60 °C. The relative viscosity is observed to be independent of the temperature. The un-solvated TiO_2_ nanoparticles are 25 nm in diameter, and the values of *K*_1_ and *K*_3_ are 0.256 and 8.5 *W* / *m* ⋅*K*, respectively. The data are plotted as 1/ η_r_ *versus* φ and as 1/ *K*_r_ *versus* φ. The plots are linear over the φ range of 0 to 0.018. The slope of 1/ η_r_ *versus* φ plot is −10.5, and the slope of 1/ *K*_r_ *versus* φ plot is −6.7. Thus, the values of [η] and [*K*] for TiO_2_-EG nanofluid are 10.5 and 6.7, respectively. Equation (24) gives a [*K*] of 2.74. Obviously, the actual enhancement of thermal conductivity of the nanofluid ([*K*]) is substantially larger than that predicted from the Maxwell equation (Equation (24)). It should be noted that these nanofluids are Newtonian in nature with the negligible dependence of viscosity on the shear rate. The shear rate range covered in the experiments is 0.5–10,000 s^−1^. The linear dependence of η_r_ on φ and the lack of shear dependence of viscosity (Newtonian behavior) suggest that the clustering of nanoparticles is negligible in these nanofluids over the φ range of 0 to 1.8%. For the given values of *K*_1_, *K*_3_, [*η*], and [*K*], we obtain the following results: *k*_s_ = 4.2, *a* = −147.5, *b* = −964.81, *c* = 707.2, *K*_2_ = 0.6653 *W* / *m* ⋅*K* and *K*_2_ / *K*_1_ = 2.6. Thus, the thermal conductivity of the interfacial layer of copper nanoparticles in TiO_2_-EG nanofluid is 2.6-times that of the thermal conductivity of the base (matrix) fluid.

Interestingly, the thermal conductivity of the interfacial nanolayers predicted from the proposed method is similar to the results of molecular dynamics (MD) simulations conducted by Liang and Tsai [22]. They found that the interfacial layers have a thermal conductivity in the range of 1.6 *K*_matrix_ to 2.5 *K*_matrix_.

## 6. Conclusions

A novel method is presented to estimate the thermal conductivity of the interfacial solvation layers surrounding the nanoparticles of a nanofluid. This information is crucial in modeling the heat transfer behavior of a nanofluid. According to the proposed methodology, the thermal conductivity of the interfacial layers of the nanoparticles can be calculated provided that the intrinsic viscosity and intrinsic thermal conductivity of the bulk nanofluid are known through experimental measurements. The thickness of the interfacial layers surrounding the nanoparticles is related to the intrinsic viscosity of the nanofluid. Thus, measurement of the intrinsic viscosity allows the estimation of the interfacial layer thickness. Once the thickness of the interfacial layer is known, the thermal conductivity of the interfacial layer is estimated from the measured intrinsic thermal conductivity of the nanofluid. The equations relating intrinsic viscosity to interfacial layer thickness and intrinsic thermal conductivity to interfacial properties (interfacial layer thermal conductivity and thickness) are developed. The proposed method of estimating the thermal conductivity of the interfacial layers of the nanoparticles is validated by simulation and experimental results.
